# Resveratrol suppresses epithelial-to-mesenchymal transition in colorectal cancer through TGF-β1/Smads signaling pathway mediated Snail/E-cadherin expression

**DOI:** 10.1186/s12885-015-1119-y

**Published:** 2015-03-05

**Authors:** Qing Ji, Xuan Liu, Zhifen Han, Lihong Zhou, Hua Sui, Linlin Yan, Haili Jiang, Jianlin Ren, Jianfeng Cai, Qi Li

**Affiliations:** 1 Department of Medical Oncology, Shuguang Hospital, Shanghai University of Traditional Chinese Medicine, Shanghai, 201203 China; 2 Research Center for Traditional Chinese Medicine and Systems Biology, Shanghai University of Traditional Chinese Medicine, Shanghai, 201203 China; 3 Shanghai Municipal Hospital of Traditional Chinese Medicine, Department of Oncology, Shanghai, 200071 China; 4 Department of Chemistry, University of South Florida, Tampa, FL 33620 USA

**Keywords:** Resveratrol, Colorectal cancer, Epithelial-to-mesenchymal transition, TGF-β1/Smads signaling pathway, Snail, E-cadherin

## Abstract

**Background:**

Resveratrol extracted from grape has been an ideal alternative drug in the therapy of different cancers including colorectal cancer (CRC). Since the underlying mechanisms of resveratrol on the invasion and metastasis of CRC have not been fully elucidated, and epithelial-to-mesenchymal transition (EMT) is a key process associated with the progression of CRC, here we aimed to investigate the potential mechanism of resveratrol on the inhibition of TGF-β1-induced EMT in CRC LoVo cells.

**Methods:**

We investigated the anticancer effect of resveratrol against LoVo cells *in vitro* and *in vivo. In vivo*, the impact of resveratrol on invasion and metastasis was investigated by mice tail vein injection model and mice orthotopic transplantation tumor model. *In vivo* imaging was applied to observe the lungs metastases, and hemaoxylin-eosin (HE) staining was used to evaluate metastatic lesions. *In vitro*, impact of resveratrol on the migration and invasion of LoVo cells was evaluated by transwell assay. Inhibition effect of resveratrol on TGF-β-induced EMT was examined by morphological observation. Epithelial phenotype marker E-cadherin and mesenchymal phenotype marker Vimentin were detected by western blot and immunofluorescence. Promoter activity of E-cadherin was measured using a dual-luciferase assay kit. mRNA expression of Snail and E-cadherin was measured by RT-PCR.

**Results:**

We demonstrated that, resveratrol inhibited the lung metastases of LoVo cells *in vivo*. In addition, resveratrol reduced the rate of lung metastases and hepatic metastases in mice orthotopic transplantation. *In vitro,* TGF-β1-induced EMT promoted the invasion and metastasis of CRC, reduced the E-cadherin expression and elevated the Vimentin expression, and activated the TGF-β1/Smads signaling pathway. But resveratrol could inhibit the invasive and migratory ability of LoVo cells in a concentration-dependent manner, increase the expression of E-cadherin, repress the expression of Vimentin, as well as the inhibition of TGF-β1/Smads signaling pathway. Meanwhile, resveratrol reduced the level of EMT-inducing transcription factors Snail and the transcription of E-cadherin during the initiation of TGF-β1-induced EMT.

**Conclusions:**

Our new findings provided evidence that, resveratrol could inhibit EMT in CRC through TGF-β1/Smads signaling pathway mediated Snail/E-cadherin expression, and this might the potential mechanism of resveratrol on the inhibition of invasion and metastases in CRC.

## Background

Colorectal cancer (CRC) is one of the leading causes of cancer-associated death in the worldwide [1]. Many patients diagnosis of CRC are at advanced stages, and the prognosis of these patients remains very poor. Generally, when the oncogenes associated with cell proliferation were up-regulated, or the tumor suppressor genes were down-regulated, the tumor cells would evade immune system and form tumors in distal locations/organs through the pathway of invasion and metastases [2,3].

Epithelial-to-Mesenchymal Transition (EMT), a biological process occurs in various types of epithelial cancers including CRC, is associated largely with increased invasion and metastases [4-6]. EMT mainly experiences the following steps: dissociation of adhesions between epithelial cells, loss of the apical-basolateral polarity, reorganization of the actin cytoskeleton, and increases of cell motility. Recently, numerous studies demonstrated that, some cytokines like TGF-β, HGF and EGF can induce the process of EMT [7-9]. Meanwhile, various signaling pathways associated with EMT were activated, such as TGF-β/Smads signaling pathway we focused on.

Traditional Chinese Medicine (TCM), whether the formula or the extracted monomer, have been identified as effective anticancer drugs in various cancers. Long-term basic research and clinical application suggested that, resveratrol has been an ideal alternative drug in the therapy of different diseases. Recently, anti-cancer activity of resveratrol has been explored in various types of cancer including pancreatic cancer, myeloma, ovarian cancer, breast cancer via the regulation of multiple pathways [10-13]. Several experimental studies have demonstrated that, resveratrol played an effective anti-cancer activity in the treatment of colorectal cancer including our own completed research [14-16].

However, the underlying molecular mechanisms through which resveratrol inhibits migration and invasion of CRC cells have not been fully elucidated, and since EMT is a key process associated with the progression of CRC, herein we aimed to investigate the potential mechanism of resveratrol on the inhibition of TGF-β1-induced EMT in CRC cells.

## Methods

### Materials

Recombinant human TGF-β1 was purchased from R&D. Resveratorl was purchased from Sigma-Aldrich and dissolved at a concentration of 100 mM in DMSO as a stock solution. Rabbit monoclonal antibodies against human E-cadherin, Vimentin, Slug, Snail, ZEB1, Twist1, MMP-2, MMP-9, β-actin were purchased from Cell Signaling Technology. Matrigel was purchased from BD Biosciences, and 24-well transwells was purchased from Corning.

### Cell culture

Human colorectal cancer cell line LoVo (ATCC, USA) was maintained in F12K medium containing 10% fetal bovine serum (FBS), 100 U/mL penicillin, 100 mg/mL streptomycin, and incubated in a humidified, 5% CO2 atmosphere, at 37°C.

### CCK assay for cell proliferation

Cell Counting Kit-8 (CCK-8) was selected to determine cell proliferation. Briefly, LoVo cells were seeded in 96-well plates at 1 × 10^4^ cells/well, when the cells reached 60% confluence, the medium was replaced with fresh medium containing different concentrations of resveratrol, and incubated for 48 h and 72 h. The medium was then discarded, and the cells were incubated with medium containing CCK-8 reagent for 4 hours. The absorbance was determined at 450 nm using a microplate reader (Biorad, USA). All the experiments were repeated three times.

### In vivo imaging by tail vein injection

Experimental lung metastases were achieved by injections of a single-cell suspension of LoVo cells containing green fluorescent protein (GFP) into the lateral tail vein. One week later, the mice were randomized into four groups of 8 animals each. Resveratrol with a dose of 0, 50, 100, 150 mg/kg [17] was interfered in distinct groups via intragastric administration every day for 3 weeks. Seven weeks later, prior to in vivo imaging, the mice were anesthetized with penobarbital sodium, and the images of established lung metastases were observed by LB983 NIGHTOWL II system (Berthold Technologies GmbH, Germany). Afterwards, both of the lung organs were excised, fixed with 10% neutral buffered formalin, and paraffin-embedded. The lung sections were fully cut, and each section was set to 6 μm. All the lung sections were stained with hemaoxylin-eosin (HE), following by counting the number of lung metastases, and assessing comprehensively the extent of metastasis. All experimental protocols were reviewed and approved by the animal ethics committee of Shuguang Hospital, Shanghai University of Traditional Chinese Medicine.

### Anti-tumor effect of resveratrol on mice with orthotopic transplantation tumor

Single-cell suspensions of LoVo cells (2 × 10^6^ in 100 μL) were injected into the subcutaneous area of female BALB/c nude mice (4–6 weeks old) obtained from SLAC (SLAC Laboratory Lab, Shanghai, China). After the tumors reached the size of 100 mm^3^, the tumors were excised, fractionated, and transplanted into the appendix of the nude mice. After two weeks, the mice were randomized into four groups of 8 animals each. Resveratrol with a dose of 0, 50, 100, 150 mg/kg [17] was interfered in distinct groups via intragastric administration every day for 3 weeks. After 42 days, animals were sacrificed by cervical dislocation in deep CO_2_ anesthesia, primary tumors were surgically removed and weighted (g). Then, a part of the removed tumors, the lung and liver of the mice were investigated by HE staining. All experimental protocols were reviewed and approved by the animal ethics committee of Shuguang Hospital, Shanghai University of Traditional Chinese Medicine.

### Western blot

Whole cell proteins were prepared according to the instructions of ProteoJET Cytoplasmic Kit (Fermentas, USA). The extracted protein was quantified by BCA protein assay. Proteins were loaded onto the SDS-PAGE gels for electrophoresis, transferred to PVDF membranes, blocked in 5% milk, and incubated with the primary antibodies following by the HRP-conjugated secondary antibodies. All the resulting immunocomplexes were visualized by enhanced chemiluminescence. Each experiment was repeated independently three times.

### Immunofluorescence microscopy

LoVo cells (2.5 × 10^5^) were fixed for 40 minutes with 4% paraformaldehyde in PBS at room temperature, blocked with 5% non-fat dry milk, and permealized with solution containing 1% BSA, 0.5% Triton X-100. The cells were first stained with E-cadherin rabbit antibody followed by Cy3-conjugated goat anti-rabbit IgG or first stained with the Vimentin rabbit antibody followed by FITC-conjugated goat anti-rabbit IgG. DAPI was applied for nuclear staining. Immunofluorescence images were taken with a DMI3000B inverted microscope (Leica, Germany). All the experiments were repeated three times.

### Transwell assay for migration and invasion

LoVo cells (5 × 10^5^, in F12K medium with 0.5% FBS) pretreated with or without different concentration of resveratrol were seeded into the upper part of a transwell chamber. For migration analysis, 600 μl F12K medium with 10 μg/ml fibronectin and 15% FBS was added in the lower part of the chamber, and the assay was performed. Migrated cells were analyzed by crystal violet staining, followed by observing under a DMI3000 B inverted microscope (Leica, Germany). Five random views were selected to count the migrated cells. For invasion analysis, 100 μl matrigel (BD, USA) was firstly added onto the bottom of the upper transwell chamber before LoVo cells were seeded, and the following procedures were as same as migration analysis, except that the invasive cells were analyzed after co-culture for 48 hours. Each experiment was repeated independently three times.

### RT-PCR

For RT-PCR of Snail gene, the primers were designed as follows: 5-CAATCGGAAGCCTAACTA-3, 5-CAGATGAGCATTGGCAGCG-3, with control GAPDH: 5-GAAGGCTGGGGCTCATTTG-3, 5-GGGCCATCCACAGTCTTC-3, and the product were confirmed with agarose electrophoresis. All assays were performed in triplicate and independently repeated three times.

### Plasmid constructions

Human Snail gene (Gene ID: 6615) was amplified by RT-PCR from LoVo cells, using the forward and reverse primers: 5-CCGCTCGAGATGCCGCGCTCTTT-3, 5-CGGGATCCTCAGCGGGGACATCC-3, sequenced by the Sangon Biotech company (Shanghai, China), and the right Snail fragments (795 bp) were sub-cloned into the expression vector of pcDNA3.1, named pcDNA3.1-Snail.

### Analysis of the E-cadherin promoter

To test E-cadherin promoter activity, LoVo cells were co-transfected with either the recombinant plasmid pGL3-basic-E-cadherin or -basic-mut-E-cadherin with a control positive plasmid pRL-SV40. The promoter activity was measured using a dual-luciferase assay kit (Beyotime Institute of Biotechnology, China) according to the manufacturer’s instructions.

### Statistical analysis

All the data were presented as mean ± standard deviation (± S) X ) and analyzed with SPSS18 Software. The mean values of two groups were compared by Student’s *t* test. *P* < 0.05 was considered as statistically significant, and *P* < 0.01 was considered as statistically highly significant.

## Results

### Resveratrol inhibited the metastases of colorectal cancer LoVo cells *in vitro* and *in vivo* imaging by tail vein injection

**Figure 1:**
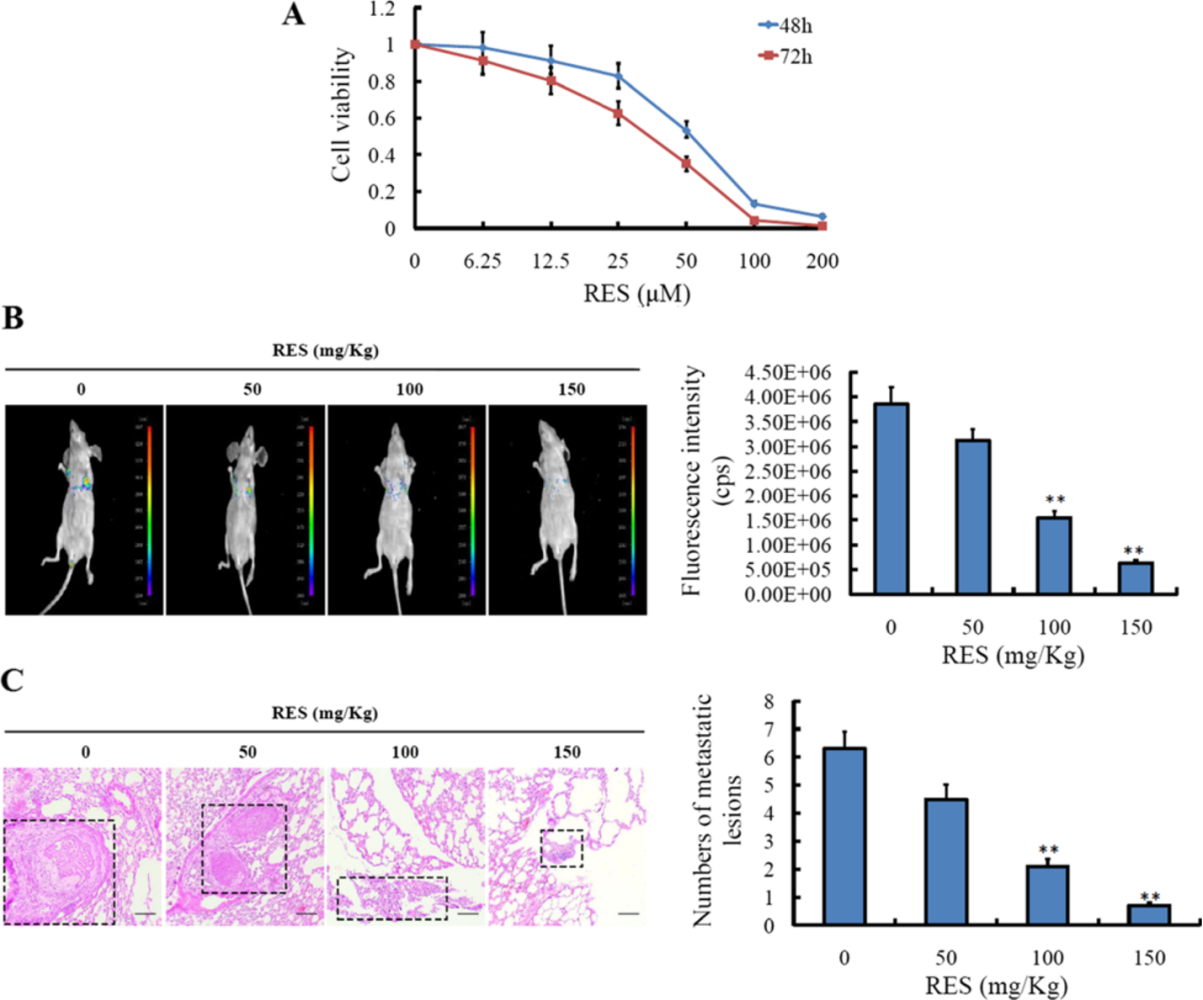
**Resveratrol inhibited the metastasis of colorectal cancer LoVo cells in vitro and in vivo imaging by tail vein injection. (A)** Correlation of resveratrol drug concnetrations (0, 6.25, 12.5, 25, 50, 100, 200 μM) and cell viability in LoVo cells for 48 h and 72 h. **(B)** LoVo-pLV4-GFP cells were respectively injected into the lateral tail vein. One week later, resveratrol was administrated with the concentration of 0, 50 mg/Kg, 100 mg/Kg, 150 mg/Kg every day for 3 weeks. Seven weeks later, the established lungs metastases images were observed by LB983 NIGHTOWL II system. **(C)** The organs of lung were excised, and the metastasis was checked by hemaoxylin-eosin staining, and the numbers of metastatic lesions were counted. **P < 0.01, compared with LoVo-pLV4-GFP cells without treatment of resveratrol.

First, we determined the cytotoxic effect of resveratrol on colorectal cancer LoVo cells using CCK assay. As shown in Figure 1A, LoVo cells were treated with various concentrations of resveratrol (0, 6.25, 12.5, 25, 50, 100, 200 μM) for 48 h and 72 h. It was observed that, resveratrol inhibited the proliferation of LoVo cells in a concentration- and time-dependent manner. After 48 h of resveratrol (12.5 μM) treatment, cell viability was reduced by approximately 10%, and this data indicated that less than 12.5 μM resveratrol had little influence on the cell proliferation of LoVo cells, while more than 50 μM resveratrol significantly inhibited the proliferation of LoVo cells (*P* < 0.01).

To observe the effect of resveratrol on LoVo cells *in vivo*, we performed the experiment of *in vivo* imaging by tail vein injection. From the results we found that, LoVo cells could partly migrate to the lung organs of the mice after tail vein injection for seven weeks, and resveratrol of different concentration (0, 50, 100, 150 mg/Kg) could inhibit the metastatic ability of LoVo cells in a concentration-dependent manner (Figure 1B). However, resveratrol has no effect on the body weight of the mice (data not shown). Then, the organs of lung were excised, and the metastatic lesions were determined by HE staining. From the imaging results we found, a lot of metastatic lesions were seen in the lung organs with no treatment of resveratrol. Nevertheless, when the increased resveratrol was treated, the metastatic lesions decreased gradually, especially at 150 mg/Kg, few metastatic lesions were found (Figure 1C).

### Resveratrol inhibited the invasive ability of the orthotopic transplantation tumor

**Figure 2:**
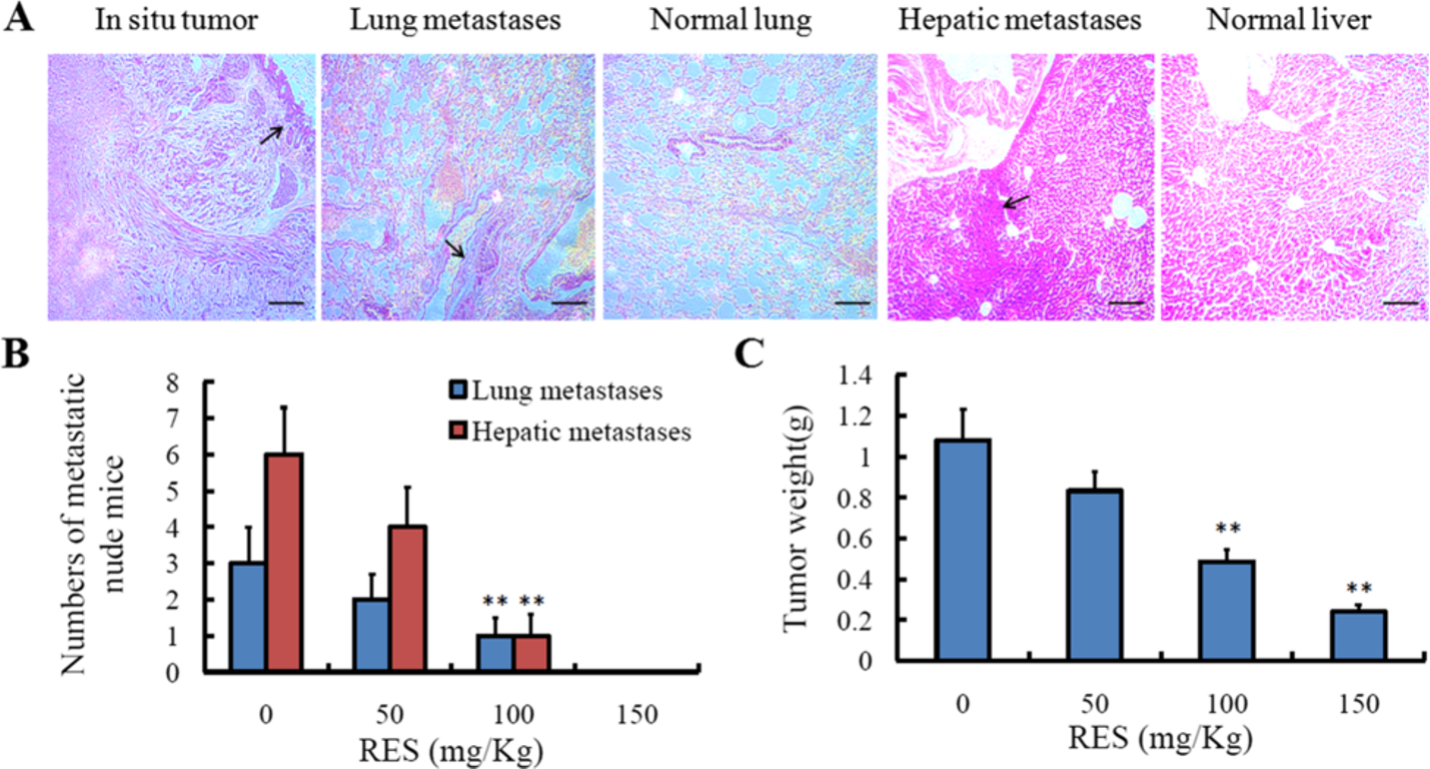
**Resveratrol inhibited the invasive ability of the orthotopic transplantation tumor. (A)** Resveratrol with a dose of 0, 50, 100, 150 mg/kg was interfered in distinct groups for the orthotopic transplantation tumor mice via intragastric administration every day for 3 weeks. After 42 days, HE staining was performed to check the numbers of metastatic lesions. **(B)** The numbers of the lung metastases and hepatic metastases were counted, **P < 0.01, compared with group without treatment of resveratrol. **(C)** The finally excised primary tumors were weighed, **P < 0.01, compared with group without treatment of resveratrol.

To investigate thoroughly the invasive ability of the orthotopic transplantation tumor originated from colorectal cancer LoVo cells, we made a long term experiment *in vivo*. As shown in the final results, the metastatic lesions were found in the district of liver and lung organs in the mice without resveratrol treatment (Figure 2A). But, with the increased treatment of resveratrol, the metastatic lesions in the liver and lung organs decreased. At 150 mg/Kg resveratrol, the metastatic lesions were rarely found (Figure 2B). Additionally, with the increase of resveratrol from 50 mg/Kg to 150 mg/Kg, the finally excised tumors decreased in a dose-dependent manner (Figure 2C), while resveratrol had no effect on the body weight of the mice (data not shown).

### Resveratrol inhibited the morphological changes of TGF-β1-induced EMT

**Figure 3:**
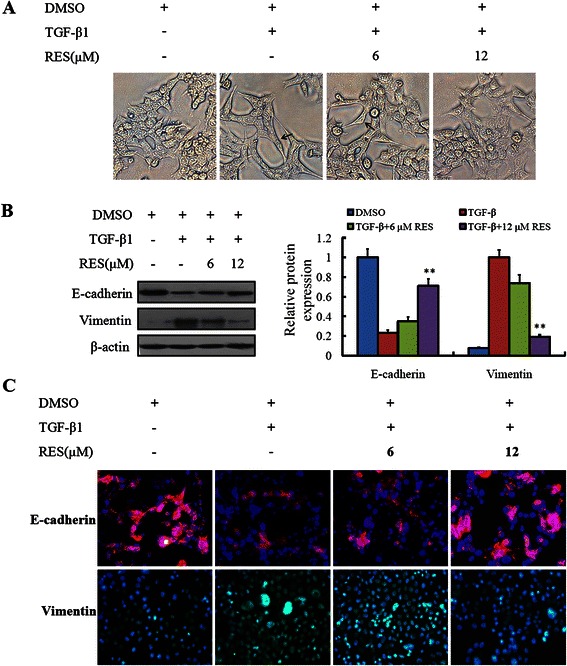
**Resveratrol inhibited the morphological changes of TGF-β1-induced EMT. (A)** LoVo cells were treated with 10 ng/ml TGF-β1 to induce EMT, and resveratrol with a concentration of 6, 12 μM were introduced to inhibit the morphological changes. Control LoVo cells displayed classical epithelial morphology, and 10 ng/ml TGF-β1 treated LoVo cells represented a mesenchymal phenotype. **(B)** Expression of epithelial phenotype marker E-cadherin and mesenchymal phenotype marker Vimentin were detected by western blot, **P < 0.01, compared with control LoVo cells without treatment of TGF-β1 and resveratrol. **(C)** Immunofluorescence staining of E-cadherin and Vimentin in LoVo cells treated with 10 ng/ml TGF-β1, with or without resveratrol.

Since resveratrol could inhibit the metastases and invasion of LoVo cells, we have enough reason to interpret the effect mechanism of resveratrol. We sought to determine whether resveratrol could inhibit TGF-β1-induced EMT in LoVo cells. The images demonstrated that, LoVo cells showed a mesenchymal phenotype after treatment with 10 ng/mL TGF-β1 for 48 h, but in the LoVo cells treated with 10 ng/mL TGF-β1 and resveratrol of 6 and 12 μM simultaneously, the mesenchymal phenotype showed fewer gradually (Figure 3A). For the characterization of EMT, we detected E-cadherin and Vimentin by western blot and immunofluorescence. From the western blot, we found, after adding TGF-β1 to induce EMT for 48 h, the expression of E-cadherin decreased obviouslywhile the Vimentin increased remarkably, and resveratrol could reverse the shifted expression of E-cadherin and Vimentin induced by TGF-β1 (Figure 3B). From the imaging of immunofluorescence we knew, after adding TGF-β1 to induce EMT, the expression of E-cadherin in the membrane decreased, but the expression of Vimentin in the cytoplasm increased obviously. But when resveratrol was added, the expression of E-cadherin and Vimentin approached gradually to the previous level without addition of TGF-β1. All above findings indicated that resveratrol could inhibit the effects of TGF-β1 on EMT in LoVo cells (Figure 3C).

### Resveratrol inhibited the migration and invasion of colorectal cancer LoVo cells treated with TGF-β1

**Figure 4:**
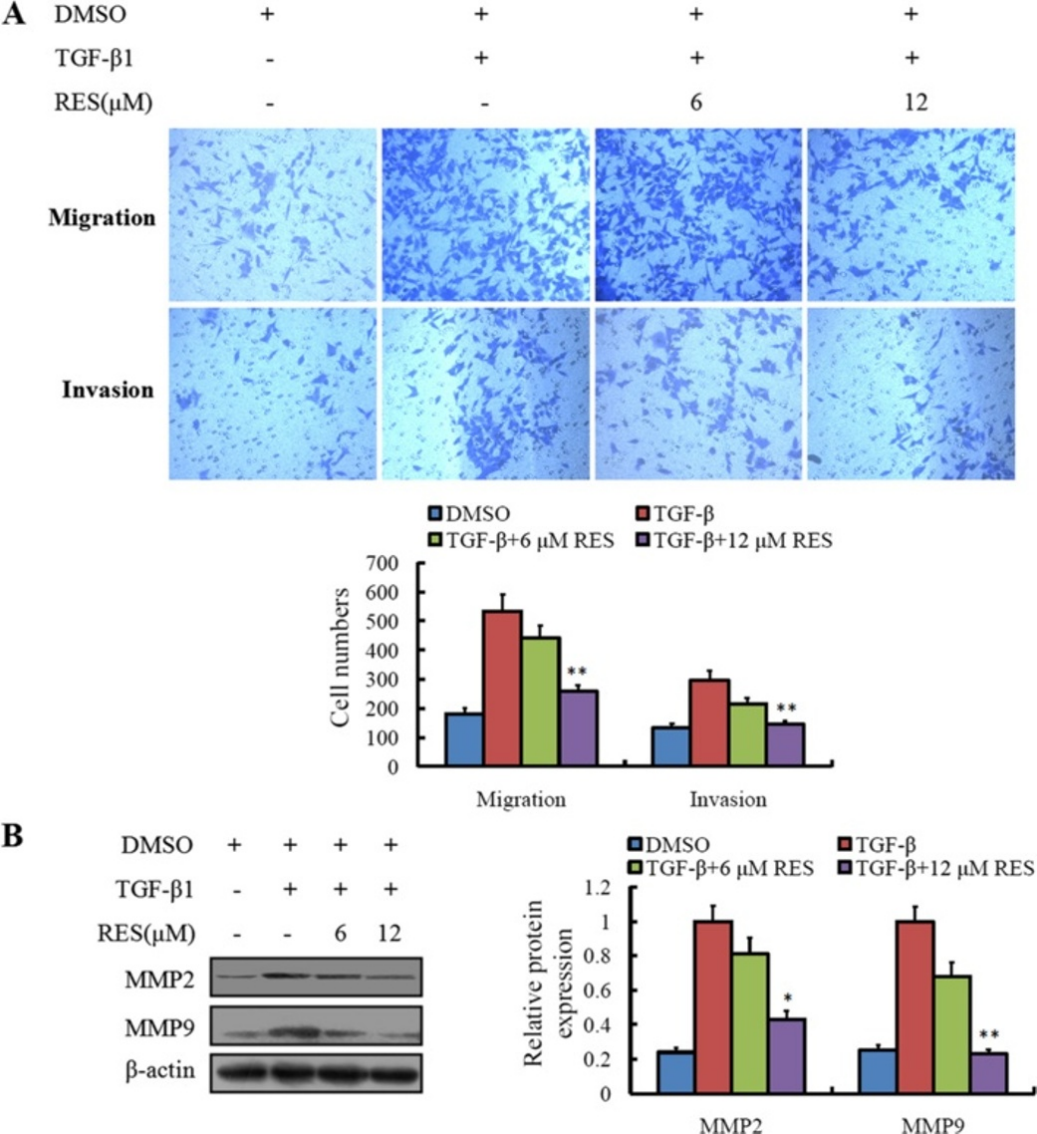
**Resveratrol inhibited TGF-β1-induced migration and invasion in colorectal cancer LoVo cells. (A)** LoVo cells treated with 10 ng/ml TGF-β1, with or without resveratrol (6, 12 μM) were applied for the trasnwell experiment. Migration and invasion of indicated LoVo cells were quantified. Values represent the number of migratory/invasive cells per 5 high power fields. **P < 0.01, compared with group without treatment of TGF-β1 and resveratrol. **(B)** MMP2 and MMP9 were determined by western blot. *P < 0.05, **P < 0.01, compared with group without treatment of TGF-β1 and resveratrol.

When the TGF-β1 induced EMT, not only the morphology of LoVo cells changed, but also their biological functions were different. By the transwell assay, we found that, being treated with 10 ng/mL TGF-β1, the migratory and invasive ability of LoVo cells increased obviously. However, resveratrol could inhibit the migration and invasion of LoVo cells caused by the TGF-β1 induced EMT (Figure 4A). Accordingly, we observed the factors of MMP2 and MMP9, which were associated closely with invasion and migration. The results demonstrated that, the expressions of MMP2 and MMP9 were consistent with the shifted ability of invasion and migration of LoVo cells (Figure 4B).

### Resveratrol repressed the expressions of EMT-associated transcription factors and inhibited the Smads signaling pathway

**Figure 5:**
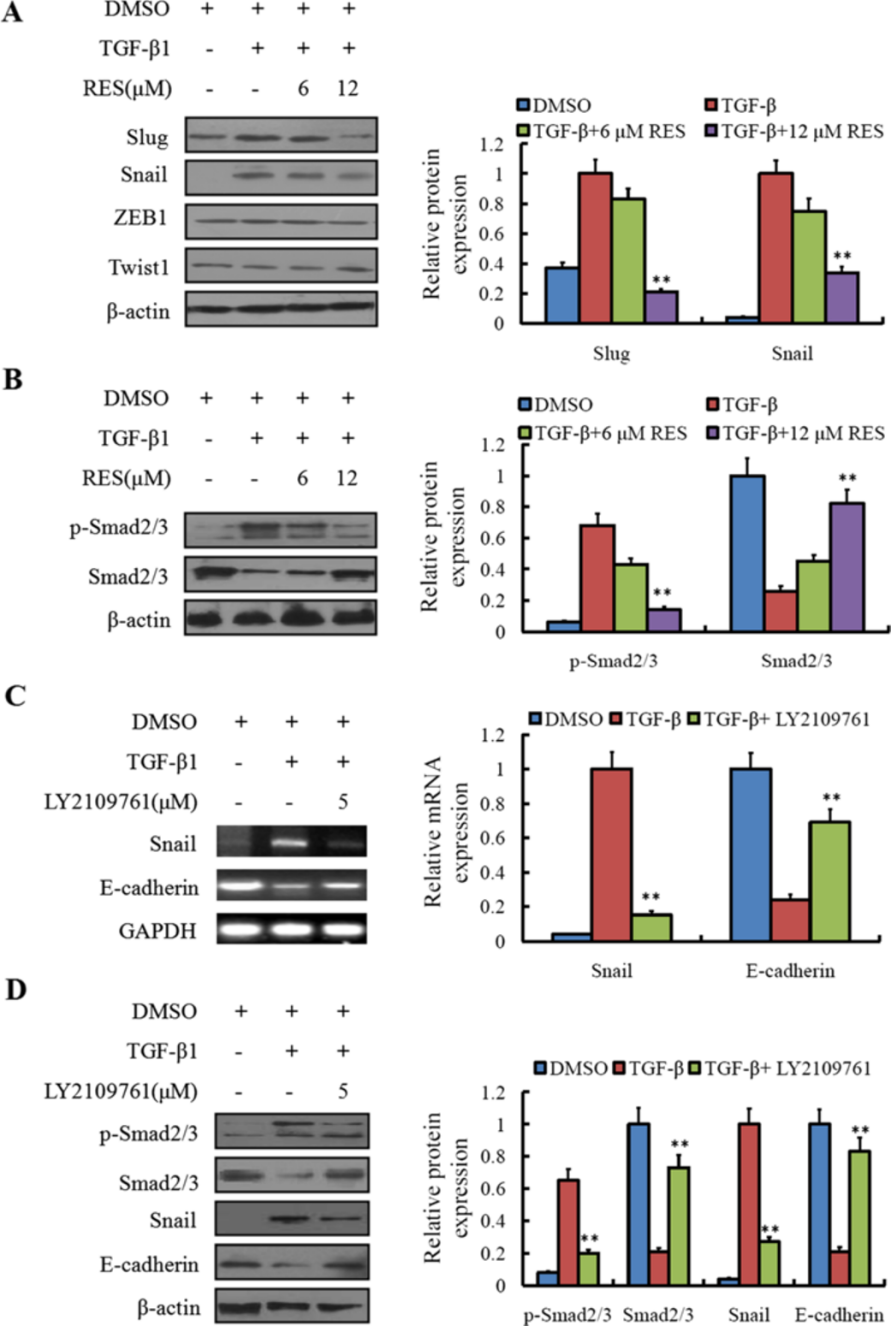
**Resveratrol repressed expressions of EMT-induced transcription factors and Smads signaling pathway.** Whole or cell extracts of different cellular compartments from LoVo cells were probed for **(A)** Slug Snail, ZEB1, Twist, and **(B)** p-Smad2/3, Smad2/3. **P < 0.01, compared with group without treatment of TGF-β1 and resveratrol. **(C)** LoVo cells treated with TGF-β1 or (and) TGFβRI/II inhibitor (LY2109761) were analyzed for the Snail and E-cadherin mRNA expression by RT-PCR, GAPDH was chosen as a control. **(D)** Whole or cell extracts of different cellular compartments from LoVo cells were probed for p-Smad2/3, Smad2/3, Snail and E-cadherin expression. **P < 0.01, compared with group without treatment of TGF-β1 and LY2109761.

To examine the inhitory ability of resveratrol on the expression of EMT-induced trascription factors, the expression of Slug, Snail, ZEB1 and Twist were detected by western blot. The results showed that, in contrast to the control LoVo cells, Snail up-regulated significantly in LoVo cells treated with 10 ng/ml TGF-β1. Nevertheless, resveratrol could inhibit the Snail level induced by TGF-β1 in a concentration-dependent manner (Figure 5A).

Considering that the TGFβ/Smad signaling pathway is a critical pathway triggered by phosphorylation of Smads, we consequently measured the active status of Smads signaling pathway. Not surprisingly, we found the expression of p-Smad2/3 elevated in TGF-β1 treated LoVo cells, and the Smad2/3 level decreased accordingly. When resveratrol was added simultaneously, the levels of p-Smad2/3 and Smad2/3 turn back to the former levels without any treatment (Figure 5B).

To confirm the effect of resveratrol on inhibiting the TGFβ/Smad signaling pathway, we used the TGFβRI/II inhibitor (LY2109761) to inhibit the TGFβ/Smad signaling pathway and observed the downstream effectors. From the RT-PCR results we knew that, when the TGFβ1 was added, the mRNA expression of Snail increased, but the E-cadherin decreased. When the TGFβRI/II inhibitor was added, the mRNA expression of Snail and E-cadherin gradually returned back to former levels, respectively (Figure 5C). Accordingly, the protein expression of Snail and E-cadherin was consistent with their mRNA expression. Additionally, the TGFβRI/II inhibitor could also inhibit the phosphorylation of Smad2/3 induced by TGFβ1 in LoVo cells (Figure 5D). The results implied that, resveratrol may regulate the EMT-associated protein expression through the TGFβ/Smad signaling pathway. To be a supplementary explanation, we also found that resveratrol had little impact on the expression of TGFβ1 or TGFβRI/II (data not shown).

### Resveratrol suppressed E-cadherin expression via inhibiting the transcription of Snail and reducing its binding on the promoter of E-cadherin

**Figure 6:**
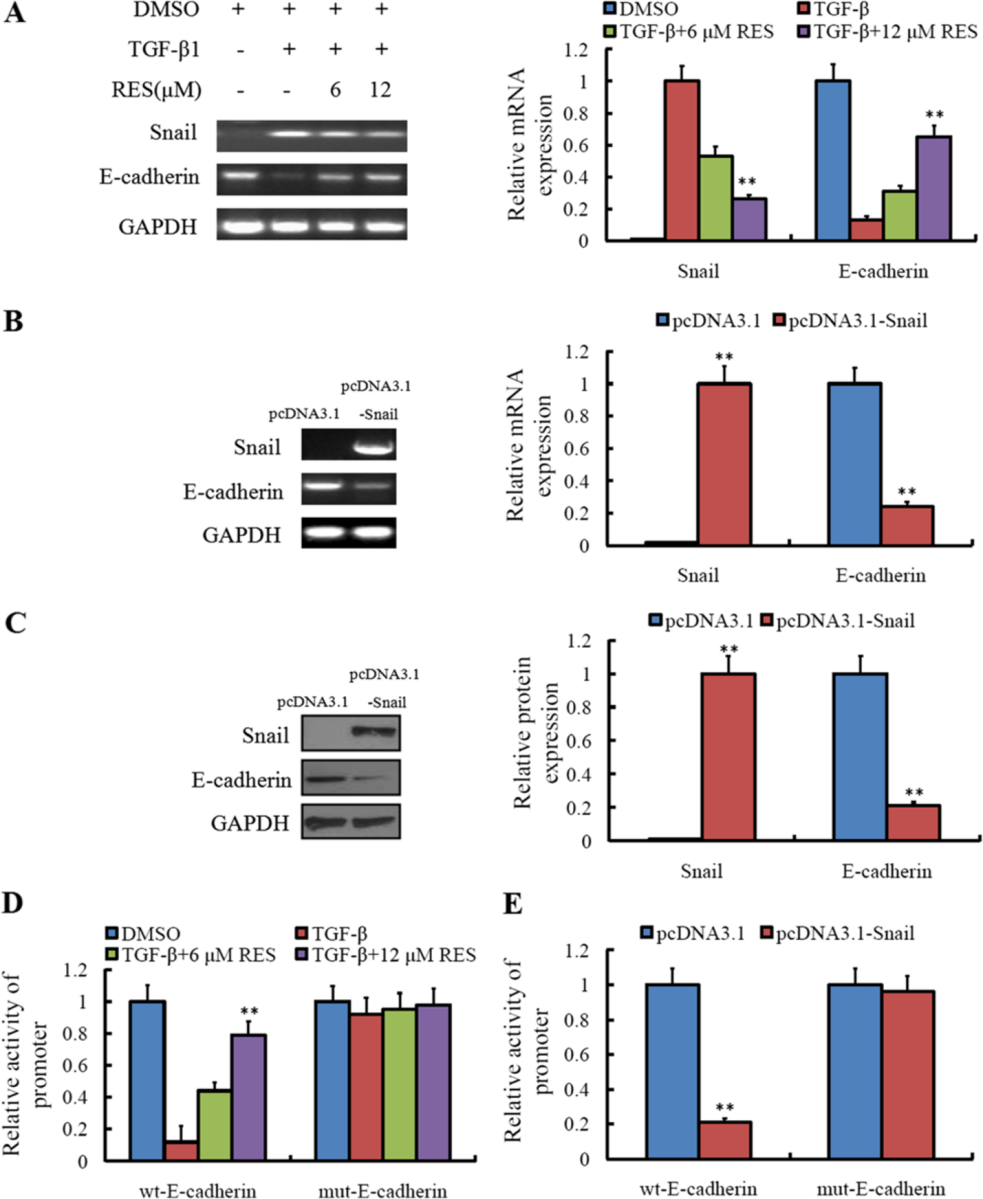
**Resveratrol suppressed E-cadherin expression via inhibiting the transcription of Snail and reducing its binding on the promoter of E-cadherin. (A)** LoVo cells treated with TGF-β1 or (and) resveratrol were analyzed for the Snail and E-cadherin mRNA expression by RT-PCR. **P < 0.01, compared with group without treatment of TGF-β1 and resveratrol. **(B)** LoVo cells trasfected with pcDNA3.1-Snail or pcDNA3.1 plasmid were analyzed for the Snail and E-cadherin mRNA expression by RT-PCR. **P < 0.01, compared with the empty vector group. **(C)** Whole or cell extracts of different cellular compartments from LoVo cells expressing pcDNA3.1-Snail or pcDNA3.1 were probed for Snail and E-cadherin. **P < 0.01, compared with the empty vector group. **(D)** The relative E-cadherin promoter activities were detected in LoVo cells treated with TGF-β1 or (and) resveratrol. **P < 0.01, compared with group without treatment of TGF-β1 and resveratrol. **(E)** The relative E-cadherin promoter activities were detected in LoVo cells expressing pcDNA3.1-Snail or pcDNA3.1. **P < 0.01, compared with the empty vector group.

In order to understand the detailed mechanism of resveratrol on EMT, we examined the effect of resveratrol on the mRNA expression of Snail and E-cadherin. As shown in Figure 6A, resveratrol could inhibit the transcription of Snail and promote the transcription of E-cadherin induced by TGFβ1 in a concentration-dependent manner. When the Snail was overexpressed in LoVo cells, the mRNA levels of E-cadherin were down-regulated obviously (Figure 6B). Accordingly, the protein expression of Snail and E-cadherin changed consistently with their mRNA levels. This indicated that Snail could affect the transcription of E-cadherin (Figure 6C). For confirming above results, we measured the effect of resveratrol and Snail on the promoter of E-cadherin. It demonstrated that, whether the resveratrol or the overexpressed Snail, they could promote the transcription activity of E-cadherin (Figure 6D and E).

## Discussions

Various plant or fruit-derived agents with few side effects have been accepted as potential alternatives for the therapy of colorectal cancer. Resveratrol extracted from grape or Polygonum cuspidatum is a natural antioxidant, which can reduce blood viscosity, maintain the blood flow, and inhibit the platelet aggregation [18-22]. In addition, resveratrol has anti-cancer activity in a great number of malignant tumors like prostate, skin, ovarian, breast and colon cancers [10-13,16].

Our previous studies have revealed the potential therapeutic effect of resveratrol against invasion and metastasis of colorectal cancer cells [16]. In that study, we found resveratrol inhibited invasion and metastasis through MALAT1 mediated β-catenin signaling pathway. Here, we observed a previously unknown mechanism, in which resveratrol could inhibit invasion and migration via reversing Epithelial-to-Mesenchymal Transition induced by TGFβ1.

EMT is characterized by the loss of cell-cell adhesion and the increase of cell motility, and it is a key process in cancer progression and metastasis [5,6], which making the inhibition of EMT process an attractive therapeutic strategy. EMT could be triggered by many growth factors like TGF-β, EGF, HGF [7-9,13,22,23]. Our present studies demonstrated that TGF-β1-induced LoVo cells undergo morphological alterations characteristic of EMT characterized by up-regulated expression of mesenchymal markers Vimentin and down-regulated expression of E-cadherin epithelial markers including and increased metastasis and invasion, up-regulated expression of mesenchymal markers Vimentin and down-regulated expression of E-cadherin epithelial markers. TGF-β1 also enhances enhanced the expression of zinc-finger transcriptional factors Snail, which then repressed the E-cadherin transcription. These transcriptional repressors of E-cadherin are required during EMT development [5]. Our study showed that resveratrol reduced migration and invasion in a concentration-dependent manner and inhibited TGF-β1-induced EMT in LoVo cells, as proved by the increase of the expression of E-cadherin and the decrease of Vimentin. During EMT development, TGF-β induced the Snail expression, and the increased Snail transcriptional factor would inhibit the promoter activity of E-cadherin, leading to the decreased E-cadherin transcription. In addition, our results showed that, the expression of EMT inducing transcription factors Snail could be inhibited by resveratrol effectively.

Although EMT is a coordinated, organized program involving interaction between different cells and tissue types, the EMT program could be activated in response to alterations of microenvironment, which would contribute to occurrence of the diseases including cancer progression [24,25]. We observed that, TGF-β1-induced EMT promoted the invasion and migration ability of LoVo cells, but reseveratrol could inhibit the promoting effect in a concentration-dependent manner. Expression analysis also demonstrated that treatment of resveratrol significantly down-regulated MMP2 and MMP9 induced by TGF-β1. Therefore, resveratrol might inhibit the invasion and metastasis of CRC cells by suppressing TGF-β1-induced EMT.

TGF-β/Smad signaling pathway is a classical pathway associated closely with the proliferation, differentiation, migration, and so on. In this system, TGF-β1 regulates cellular processes by binding and phosphorylating cell-surface receptors (TGF-βRI/TGF-βRII), and the activated TGF-βRI phosphorylates Smad2 or Smad3, will bind to Smad4 [26,27]. The resulting Smad complex then moves into the nucleus, where it interacts in a cell-specific manner with various transcription factors to regulate the transcription of many genes [23,28]. Snail was one of TGF-β/Smad signaling pathway mediated gene [29,30], which repressed the E-cadherin expression, promoted the EMT process, and finally increased the ability of invasion and metastasis of CRC cells in our study. However, resveratrol could inhibit the invasion and metastasis by preventing the continuation of EMT process.

## Conclusions

**Figure 7:**
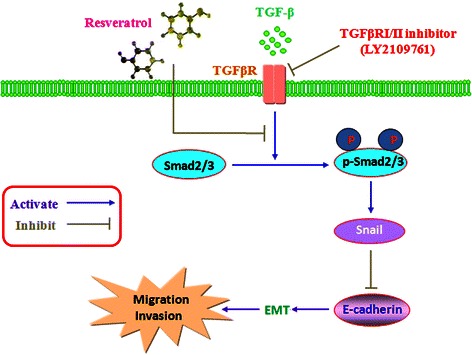
**A hypothetical illustration for the mechanism of resveratrol on the EMT of colorectal cancer.**

In summary, our study provided evidence that resveratrol could inhibit EMT in colorectal cancer through TGF-β1/Smads signaling pathway mediated Snail/E-cadherin expression, and this might the potential mechanism of resveratrol on inhibition of invasion and metastases (Figure 7).

## Abbreviations

CRC: Colorectal cancer
EMT: Epithelial-to-mesenchymal transition
HE: Hemaoxylin-eosin
TCM: Traditional Chinese Medicine
CCK-8: Cell Counting Kit-8
GFP: Green fluorescent protein
FBS: Fetal bovine serum
